# Blood-based Biomarkers for Early Diagnosis of Lung Cancer: A Review Article

**DOI:** 10.31729/jnma.5023

**Published:** 2020-07-31

**Authors:** Narayani Maharjan, Niresh Thapa, Jiancheng Tu

**Affiliations:** 1 Program and Department of Clinical Laboratory Medicine, Center for Gene Diagnosis, Zhongnan Hospital of Wuhan University, Wuhan, China; 2 Karnali Academy of Health Sciences, Jumla, Nepal

**Keywords:** *biomarkers*, *blood*, *early diagnosis*, *lung neoplasms*, *microRNAs*

## Abstract

Lung cancer is the severe leading cause of cancer-related mortality worldwide. Early detection of lung cancer can significantly increase their survival rate. However, conventional lung cancer screening methods such as sputum cytology, chest X-rays, low-dose computed tomography, positron emission tomography, and magnetic resonance imaging, are radiational, and also expensive methods. Similarly, lung tumor tissue as invasive and difficult to obtain and potentially risky procedures, there is the immediate need of non-invasive, novel sensitive and reliable blood-based tumor markers which now has become an important area on research. This review will mainly focus on recently identified circulating biomarkers: circulating tumor cells, circulating tumor deoxyribonucleic acid, tumor-derived exosomes, circulating ribonucleic acid and micro ribonucleic acid, and tumor-educated platelets which may enable earlier diagnosis of lung cancer and their application in clinical practices.

## INTRODUCTION

Lung cancer (LC) is the most commonly occurring malignancy and the leading cause of death worldwide in both genders. According to GLOBOCAN 2018, LC was the major cause of cancer incidence and mortality worldwide, with 2.1 million new LC cases and 1.8 million deaths in 2018, representing close to 1 in 5 (18.4%) deaths by cancer.^1^

LC typically comprised of two major categories: nonsmall cell lung cancer (NSCLC) and small-cell lung cancer (SCLC), accounts for 85% and 15% of LC, respectively.^2^ LC has among the worst five-year survival of any cancer which is 18%.^3^ The early detection of cancer can greatly reduce the probability of distance metastasis, thereby improving the survival rate of cancer patients. But the lack of efficient early detection methods and the ability to cure metastatic disease attribute to the poor prognosis of LC. The objective of this review is to explore recent advances in blood-based biomarkers for early diagnosis of LC.

## EARLY DETECTION OF LUNG CANCER

Typically, LC is first detected from a standard chest X-ray or Computed tomography (CT) scan. The data from the National Lung Screening Trial showed that screening with CT scans decreased LC mortality by 20% relative to screening with chest X-rays.^4^ Early diagnosis of LC has been proved to be very challenging. LC screening with low-dose CT scans often detects small lung nodules, or lesions, that cannot be diagnosed as clearly benign or cancerous which usually require follow-up with repeated CT scans, a biopsy, or even surgery.^5^ As LC incidence and mortality are increasing worldwide, better diagnostic methods are immediately required to improve the detection of LC.

Nowadays, for the early detection of LC, tissue and blood have been extensively used. Tumor-tissue derived diagnosis has been the gold standard, and biomarkers derived from tumor tissue have been the main spotlight in establishing prognostic and predictive markers in LC.^6^ However, tissue biopsies are invasive, and reliance on tumor tissue has apparent shortcomings as it does not allow for serial sampling of the tumor over the treatment course, nor does it allow for multiple biopsy attempts to assess intra-tumor heterogeneity.^6,7^ Blood-based biomarkers are relatively non-invasive having the potential of assessing real-time tumor response to therapy and identifying dynamic resistant clones. As new technologies develop, they are becoming faster and more accurate.^7^ Also, blood-based screening using circulating tumor cells (CTCs), circulating Deoxyribonucleic acids (ctDNAs), circulating Ribonucleic acids (ctRNAs), and extracellular vesicles (EVs) have shown promising prospects in the early detection of cancer. Recent high-throughput gene expression profiling has provided a valuable resource for developing new biomarkers for the early detection of cancer.^8^

## BLOOD-BASED BIOMARKERS FOR LUNG CANCER

Blood, a complex and dynamic medium, whose components can indicate various physiologic or pathologic states such as the presence of some cancers.^9^ CTCs and macromolecules such as proteins, lipids, Deoxyribonucleic acid (DNA), Ribonucleic acid (RNA), and microRNA (miRNA); that derive from tumor cells can be found in the blood.^10^

A biomarker is defined as “a characteristic that is objectively measured and evaluated as an indicator of normal biological processes, pathogenic processes, or pharmacologic responses to a therapeutic intervention".^11^ The ideal cancer biomarker must be useful for all purposes (diagnosis, prognosis, or the prediction of pharmacological responses). It should be easy to obtain, easy to perform, inexpensive, accurate, and highly informative.^10^ Blood in particular, with its cellular, micro-particulate, and plasma constituents, represents a rich source of biomarkers.^12^ Recently different reviewed done by Mamdani et al. and Welliver et al. focused on various blood-based biomarkers that have been recognized such as CTCs, circulating cell-free nucleic acids, such as ctDNA and miRNA; tumor-educated platelets (TEPs), tumor-derived exosomes; and other biomarkers such as genomic and proteomic features and these review paper also highlight the existing evidence for their use in prognosis, detection, and treatment decision of LC.^6,7^ An analysis of the most recent publications in early diagnosis of biomarkers appears that the target in cancer biomarkers has altered from searching for single biomarkers to evaluating combined biomarkers, which sounds to be more effective in decreasing false positives and increasing detection rates.

The European Group on tumor markers has recently presented guidelines (MONITOR) for designing studies on the validity of tumor markers for the serial monitoring of cancer patients, to show whether monitoring improves outcomes, compared with other routinely used methods.^13^ The growth of the tumor is accompanied by changes at the DNA, RNA, miRNA, and protein levels which can be used for the discovery and clinical evaluation of prognostic and predictive biomarkers for LC.^14^ Epigenetic markers such as DNA methylation, miRNAs, nucleosome remodeling, and histone modifications have also been investigated.^9^ Recently many studies done on blood biomarkers suggest that circulating biomarkers have the best potential to be a cost-effective method for early LC detection.

## CURRENTLY AVAILABLE BLOOD-BASED BIOMARKERS IN LUNG CANCER CIRCULATING TUMOR CELLS (CTCS)

CTCs are rare cells originating from the malignancy that circulate freely in the peripheral blood.^9^ Tumor-derived cells identified in peripheral blood can be used for the diagnosis of original cancer or the detection of recurrent cancer.^15^ Although enormously rare, CTCs show a potential alternative to invasive biopsies as a tumor tissue source for the detection, characterization, and monitoring of non-hematologic cancers.^16^ Nowadays, various techniques have been developed for the isolation, characterization, and enumeration of CTCs and these multiple techniques show great variability in detection rates, sensitivity, and specificity of CTC.^17^ CTCs are mostly captured by immobilized anti-epithelial cell adhesion molecule (EpCAM, also known as TACSTD1) antibodies either in chip or bead platforms.^15^ The CellSearch (Veridex) system is one of the most validated and only United States Food and Drug Administration (US FDA) approved the method of obtaining CTCs till date.

In a cohort study of 101 patients with NSCLC, by the use of the CellSearch System, the number of CTC was a strong predictor of overall survival (OS).^18^ Patients with CTCs greater or equal to 5 per 7.5 ml blood are considered to show a lack of response to treatment while those with <5 CTC have been considered to show remission.^19^

As CTCs typically present with non-epithelial characteristics, CTCs detection in LC has been challenging.^20^ This emphasizes the call for more sensitive technologies to better capture CTCs for indepth characterization and functional studies using cell culture and xenograft models.^21^ Rare CTCs capture technology is still in the early phase of development and requires more specific surface markers to raise its specificity for circulating LC cells.^9^

## CIRCULATING TUMOR DNA (CTDNA)

ctDNA is the subset of cell-free DNA (cfDNA) coming from tumor cells to the bloodstream, and these DNA fragments contain the complete genome of primary tumor tissue. Hence, ctDNA is a reliable alternate for tumor tissue.^22^ Even though it was first identified in 1977 but has gained more relevance only recently in the last decade as gene sequencing technologies became faster, cheaper, and more accurate.^7^

ctDNA consists of short fragments of double-stranded DNA of approximately 160-180 bp^23^ which is likely released from tumor cells by necrosis, apoptosis, or secretion^24^ via exosomes, and hence, ctDNA contains tumor-specific sequences that harbour the somatic genomic alterations found in tumor tissue.^25^

Sanger sequencing is a traditional approach to ctDNA, lack sensitivity and a more appropriate for DNA with longer reads, which makes these methods inadequate for ctDNA analysis. Recently, there are multiple highly sensitive and specific platforms for ctDNA detection mainly based on Polymerase Chain Reaction (PCR) or next-generation sequencing (NGS).^22^ The BEAM (beads, emulsion, amplification, magnetics) technology and CAPP-seq (cancer personalized profiling by deep sequencing) are the new technologies that have changed the scenery of ctDNA. These methods amplify target DNA using already known tag sequencing primers. CAPP-seq can even quantify ctDNA and can also identify mutations in 100% of stage II-IV and 50% of stage I NSCLC patients, indicating its prognostic value and specificity was 96%.^26^ Some studies have found that ctDNA in plasma of NSCLC patients is higher than healthy control subjects and some other studies found higher circulating DNA levels in NSCLC patients with lymph node metastasis or distance metastasis, and also that patients had less OS time. These findings suggested that circulating DNA can act as a potential diagnostic marker for NSCLC.^27^ Epidermal growth factor receptor (EGFR) mutation status is an important biomarker for NSCLC targeted therapy so it is helpful to have an early diagnosis about mutation type for getting optimal targeted therapy. And some studies have shown that ctDNA were highly consistent, an important biomarker for cancer prognosis and is capable of detecting EGFR mutations in ctDNA of patients with NSCLC.^28–9^

## CIRCULATING RNA AND MICRORNA

MicroRNAs are small, non-coding RNA molecules, 20-25 nucleotides in length that negatively regulate gene expression at the post-transcriptional level.^29^ Nowadays circulating microRNA has become a major research area and there is a burgeoning concern in analyzing it as a non-invasive biomarker for NSCLC diagnosis, monitoring response to treatment, and potentially to personalize therapy.^7^ Researches showed that circulating miRNA become dysregulated during tumor development and thus result in abnormal miRNA profiles in cancer patients.^30^ Different studies have shown that circulating miRNAs could be considered as a novel promising biomarker for early detection and screening of LC because (a) serum miRNAs are readily detectable by reverse transcription quantitative realtime PCR (RT-qPCR) technique which is broadly used in clinical laboratories and (b) as blood-based biomarkers are minimally invasive for the screening of high-risk subjects and early diagnosis of LC.^31^

A study done by Shen et al. demonstrated the diagnostic value of a set of four miRNAs, including miRNA-21, miRNA-126, miRNA-210 and miRNA-486-5p, which was evaluated by real-time quantitative reverse transcription PCR (Real-time qRT-PCR) assay and whose altered expression had been previously validated in lung tumor tissues, yielded 86.22% sensitivity and 96.55% specificity in distinguishing patients of NSCLC from healthy controls.^32^ Another study conducted by Heegaard, et al. with the use of qRT-PCR to evaluate and compare the expression levels of 30 selected miRNAs in a larger sample size of early-stage NSCLC patients and controls in serum and plasma. The study reported an increase in serum expression of miR-29c in NSCLC patients, while a set of 7 miRNAs (miR-146b, miR-155, miR-221, miR-17-5p, miR-27a, miR-106a, and let-7a) was significantly reduced in the serum of NSCLC cases. Here in this study, overall expression levels in serum did not correlate well with levels in plasma in which no significant differences of miRNAs levels were observed between patients and healthy controls, indicating these two sources may not be comparable regarding miRNA expression.^33^ Numerous miRNAs also play significant roles in the pathogenesis of LC and have the potential to be diagnostic markers and therapeutically-targeted molecules. All these data and studies suggest a putative role of miRNAs, therefore genome-wide screening can help to identify novel cancer-associated miRNAs which could serve as diagnostic markers or cancer recurrence indicators.^34^

## EXOSOMES

Exosomes are extracellular vesicles of 40-100 nm in size which are released by several cell types, along with cancer cells, into the extracellular space and a variety of body fluids.^35^ One of the most enticing aspects of exosomes research is the discovery of novel biomarkers for the early and improved detection of LC.^36^ Many studies proved that exosomes have a vital role in cell-cell communication as they transfer information (including proteins, DNA, and RNA) to target cells through fusion with the plasma membrane, receptor-ligand interaction with the cell or endocytosis by the phagocytic mechanism.^7,35^ They have been demonstrated a key role in tumor biology including local growth, metastasis, and drug resistance by transferring oncogenic proteins and nucleic acids to the tumor cells. Thus, exosomes and their content could potentially serve as important biomarkers in prognosis, diagnosis, and prediction of response to treatment.^37^

Over the past decade, many advanced techniques have been used and impressive advances have been made in the development of novel exosome isolation methods, such as Magnetic activated cell sorting (MACS), sucrose gradient method, and ultra-centrifugation, including extracellular vesicle array or immune beads precipitation.^38^ Once accumulated from a biological fluid, Western Blot or ELISA, quantitative RT-PCR, nucleic acid sequencing, and other commercially available kits can be applied to characterize the RNA and exosomes protein contents.^39^ Unlike circulating microRNAs, exosomal microRNAs are enriched in the circulatory system and protected from RNase degradation.^35^

The circulating exosomes' level and their contents (RNA and miRNAs) have been examined to explicate their potential role in guiding diagnosis and predicting prognosis of patients with lung adenocarcinoma. A study done by Cazzoli, et al. has reported a model based on microRNAs derived from circulating exosomes capable to differentiate between lung adenocarcinoma and granuloma. Besides, found a potential screening and diagnosing value of exosomal miRNAs for lung adenocarcinoma.^40^ Exosomes can be isolated in cancer patients from other body fluids, including pleural effusions.^41^ Recently, Tamiya et al. have shown that two exosomal miRNAs (miR-182 and miR-210) in the pleural fluid may serve as promising biomarkers for the diagnosis of malignant pleural effusions from lung adenocarcinoma over benign pleural effusions with the area under the curve (AUC) values of 0.87 and 0.81 respectively.^42^ Hence, it could be researched for other biomarkers, including oncogenic alterations, possibly effective for a wider range of clinical applications.

## TUMOR-EDUCATED PLATELETS (TEPS)

TEPs are blood platelets that contain tumor RNAs and they are a great source of tumor-derived RNAs.^43^ Platelets, known for their hemostasis role, interact with tumor cells and affect tumor growth, invasion, and establishment of distant metastasis. Tumor-associated biomolecules transfer to the platelets via confrontation with tumor cells results in sequestration of such biomolecules giving rise to TEP.^44^ Studies have shown that platelet size and platelet count can already provide clinically relevant information about the presence of cancer.^45^ It has been indeed shown that tumor-derived platelet factor 4 (PF4, CXCL4) promotes bone marrow megakaryocyte-mediated platelet production in patients with NSCLC.^46^ Further, platelets go through specific splicing of pre-mRNAs in response to activation of platelet surface receptors, which gives rise to a unique mRNA profile which can be potentially utilized for cancer diagnostics.^44,47^

TEPs are associated with the progression and spread of several solid tumors, and spliced TEP RNA surrogate signatures can provide specific information on the presence, location and molecular characteristics of cancers. Here to fore, TEP samples from patients with different tumor types, including lung, breast, and brain cancers, have been tested, and it has been shown that TEPS from patients with cancer is discrete from those with inflammatory and other noncancerous diseases.^48^ A study was done by Best MG, et al. comparing the platelet mRNA profiles of cancer patients with those of healthy controls indicated that the levels of 20 nonprotein-coding RNAs were altered in TEPs as compared to platelets from healthy individuals.^48^ Among the 5003 RNAs detected in all samples, a total of 1,453 of mRNAs showed to be increased (concerning hypoxia, differentiation, and immunodeficiency pathways) and 793 were decreased (concerning translation, RNA processing, and viral replication) in TEPs as compared to platelet samples from healthy donors. Moreover, TEPs mRNA profiles allowed differentiation of patients with Kristen rat sarcoma virus (KRAS) mutant tumors from KRAS wild-type tumors, EGFR mutant tumors, and MET overexpression in patients with NSCLC. However, they were unable to measure significant differences between non-metastasized and metastasized tumor, consequently implying these TEP mRNA profiles do not have the power to discriminate between certain stages of cancer and also analyzed samples number was somewhat small along with the risk of algorithm overfitting.^47^ Nevertheless, this study provides an early sign that TEPs can be potentially applied as a blood-based biomarker of LC in the future.

In conclusion, blood-based biomarkers, non-invasive with several clinical advantages compared to tissue biomarkers could potentially be effective biomarkers in early diagnosis of LC. Nowadays, with the development of new and highly sensitive technologies, it can be useful tools for routine clinical practice, including early diagnosis of LC as a potentially curable disease. Currently available blood-based markers surely provide an efficient surrogate of tissue biopsy in patient population and appear assuring in providing a new aspect to personalized cancer care.

## Conflict of Interest

**None.**
